# Osteochondral organoids: current advances, applications, and upcoming challenges

**DOI:** 10.1186/s13287-024-03790-5

**Published:** 2024-06-21

**Authors:** Maryam Faeed, Mahsa Ghiasvand, Bahar Fareghzadeh, Leila Taghiyar

**Affiliations:** 1 Cell and Molecular School of Biology, College of Science, University of Tehran, Tehran, Iran; 2 Department of Animal Sciences and Marine Biology, Faculty of Life Sciences and Biotechnology, Shahid Beheshti University, Tehran, Iran; 3 Department of Biomedical Engineering, Science and Research Branch, Islamic Azad University, Tehran, Iran; 4 Department of Stem Cell and Developmental Biology, Cell Science Research Center, Royan Institute for Stem cell Biology and Technology, ACECR, Tehran, Iran; 5 Advanced Therapy Medicinal Product Technology Development Center (ATMP-TDC), Cell Science Research Center, Royan Institute for Stem Cell Biology and Technology, ACECR, Tehran, Iran

**Keywords:** Osteochondral Organoid, Stem cells, 3D culture, Mini-joint, Regenerative orthopedics

## Abstract

In the realm of studying joint-related diseases, there is a continuous quest for more accurate and representative models. Recently, regenerative medicine and tissue engineering have seen a growing interest in utilizing organoids as powerful tools for studying complex biological systems in vitro. Organoids, three-dimensional structures replicating the architecture and function of organs, provide a unique platform for investigating disease mechanisms, drug responses, and tissue regeneration. The surge in organoid research is fueled by the need for physiologically relevant models to bridge the gap between traditional cell cultures and in vivo studies. Osteochondral organoids have emerged as a promising avenue in this pursuit, offering a better platform to mimic the intricate biological interactions within bone and cartilage. This review explores the significance of osteochondral organoids and the need for their development in advancing our understanding and treatment of bone and cartilage-related diseases. It summarizes osteochondral organoids’ insights and research progress, focusing on their composition, materials, cell sources, and cultivation methods, as well as the concept of organoids on chips and application scenarios. Additionally, we address the limitations and challenges these organoids face, emphasizing the necessity for further research to overcome these obstacles and facilitate orthopedic regeneration.

## Introduction

Several joint-related diseases decrease individuals’ quality of life and impose a substantial burden on societies and healthcare systems worldwide [1], including non-union fractures [2], osteosarcoma [3], osteoporosis [4], osteoarthritis (OA) [5], ankylosing spondylitis [6], gout [7], and rheumatoid arthritis (RA) [8]. In these pathological conditions, mainly OA, the structure of the joint is altered, and the cartilage and subchondral bone go through degradation and remodeling, respectively [9, 10]. An osteochondral unit that contains articular cartilage and subchondral bone, covers the joint surface and is responsible for its movement and transmission of load-bearing weight over it [11]. Hence, investigating its structure and composition can aid in joint disease.

On the one hand, cartilage with its limited self-repair ability makes treatments challenging and inadequate [12]. On the other hand, bone, as a main section of the joint, is capable of regenerating its minor defects. However, this self-repair is ineffective for more extensive fractures and remains an obstacle for orthopedic physicians [13]. In this regard, tissue engineering methods in regenerative medicine may be an efficient alternative for osteochondral diseases and injuries. Developing a tissue-engineered system to study the development of joint, and related pathological conditions and drug monitoring is utterly beneficial [14]. Therefore, “osteochondral organoids” emerged as in vitro models to increase our knowledge of the interaction between those two tissues in both physiological and pathological conditions.

The term “organoid” refers to a three-dimensional culture system derived from stem cells or tissue-resident progenitor cells that captures the complex architecture, cellular composition, and functionality of the modeled tissues [15]. Their possible self-renewal and self-organization capability can make them physiologically relevant models for developmental biology, disease modeling, and drug testing in vitro [16]. To date, organoids have been successfully generated for various organs such as kidney [17], intestine [18], colon [19], brain [20], and liver [21].

**Fig. 1:**
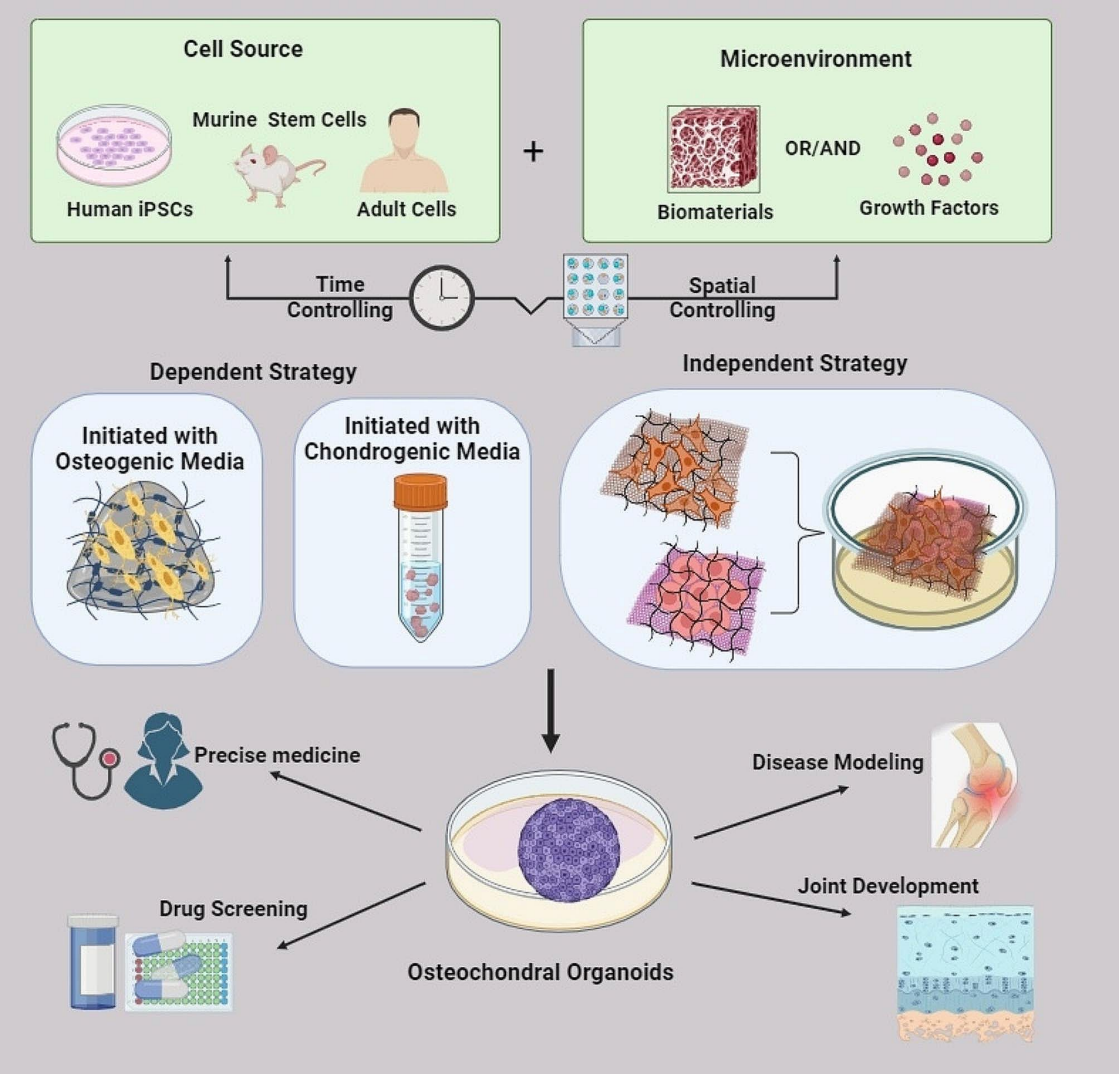
Graphical abstract. This is a summary that explains the osteochondral organoid strategies with their application

While bone and cartilage organoids have been developed to enhance our understanding of joint diseases, they do have limitations when replicating the complex hierarchical structure and interactions between different cell populations found in natural joints [22, 23]. Osteochondral organoids emerged as advantageous models in the regenerative orthopedic field by mimicking the origin environment and by incorporating different cell types, such as chondrocytes and osteoblasts, to better reflect the complex interactions between different cell populations within the osteochondral unit [24]. Moreover, these organoids can be personalized using patient-derived cells, allowing for studying individual-specific disease mechanisms and personalized medicine approaches [25]. They also open up new possibilities for regenerative medicine, as they may potentially be transplanted into damaged joints to induce tissue repair and regeneration. Here in, we will introduce joint structure characteristics, and summarize the cell sources and material for the generation of osteochondral organoids models, their application prospects, and the current shortcomings in this field Fig. 1.

## Structure of osteochondral unit

**Fig. 2:**
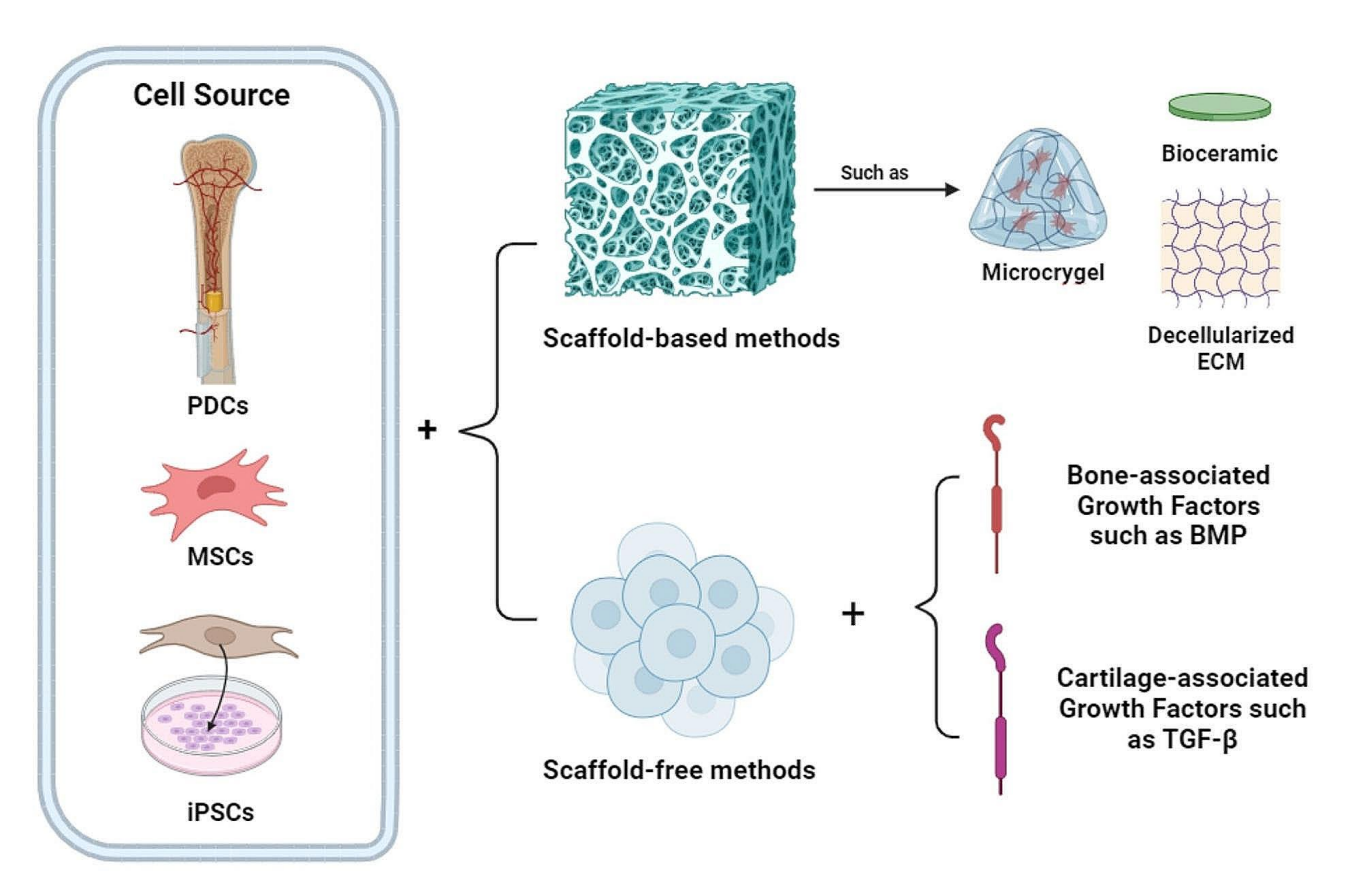
Organizing cell origins and microenvironment for osteochondral organoids. PDCs: periosteum-derived cells, MSCs: mesenchymal stem cells, iPSCs: induced pluripotent stem cells, CM: extracellular matrix, BMP: bone morphogenic protein, TGF: transforming growth factor

The synovial joint emerges as a central player in various pathophysiological processes, particularly in osteochondral (OC) regeneration. OC unit constitutes a sophisticated structure crucial for facilitating joint motion and maintaining flexibility [26]. This joint is enveloped by a synovial membrane [27] and comprises two primary elements that originate from the mesoderm layer during embryonic development: the articular cartilage and the subchondral bone [28, 29] (Fig. 2). The joint cavity is filled with synovial fluid that contains signaling factors and provides nutrients for avascular cartilage [30]. Moreover, a capsule of ligaments and tendons surrounds the joint, contributing to its stability [31]. Emerging insights into the OC interface, the region between subchondral bone and hyaline cartilage, underscore its significance in maintaining joint structural integrity [32]. The crosstalk between cartilage and subchondral bone components makes the joint a complex functional unit [33]. Diffusion and vascular channels facilitate the communication between these two sections. The vascularization in the bone matrix affects the mediators produced by both bone and cartilage sections and affects OC units [34]. These interactions are necessary for osteochondral unit development and their alteration will impact joint pathobiology [11]. The proximity of its layers allows for the maintenance of homeostasis through precisely controlled regulatory pathways, enabling effective molecular and biochemical communication between tissues and adaptive responses to environmental cues [35]. The present cells in the joint including chondrocytes, osteocytes, synoviocytes, synovial fibroblasts, and tissue-resident macrophages produce transcription and growth factors to modulate the interaction between the cell-cell and cell-microenvironment [36].

Moreover, OC units are prone to various conditions and injuries, such as OA [37]. Therefore, understanding their structure and interactions is essential for diagnosing and treating disorders, as well as for developing strategies for joint tissue engineering.

### Structure of articular cartilage

**Fig. 3:**
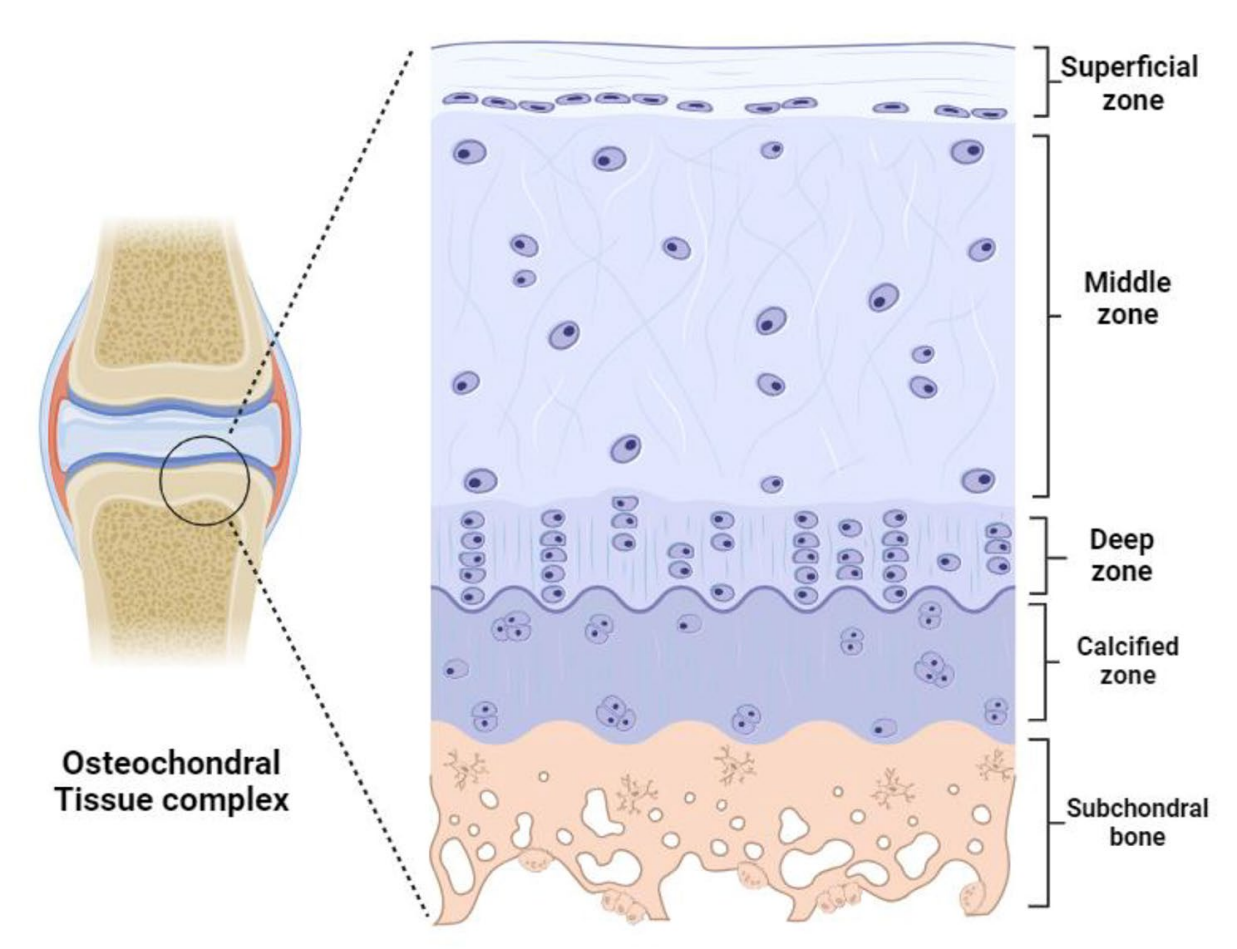
Microstructure of osteochondral tissue complex

Articular cartilage is located on the external boundary of movable joints and serves as a superficially lubricated cushion that minimizes friction between adjacent bones [38]. This avascular connective tissue plays a vital role in the mechanical loading transition into the deep subchondral bone plate while facilitating smooth bone movement [39]. The articular cartilage’s unique structure and functions make it a key determinant of joint health, and its role in OC unit dynamics is indispensable [40]. During embryonic development, articular cartilage is derived from the mesoderm [41] and exhibits a nuanced macro and microstructure, consisting of four distinct zones: calcified, deep, middle, and superficial zone (Fig. 3) [42]. The thin layer of articular cartilage comprises chondrocytes, dense extracellular matrix, and fluid-filled spaces known as lacunae [43]. The extracellular matrix itself is a complex biochemical microenvironment that contains various proteins and glycosaminoglycans, which regulate the functions of many cells and affect the stiffness and load-bearing of cartilage [44]. This organization contributes to the overall biomechanical properties of the joint. There are several diseases related to articular structure. However, when the articular cartilage is damaged, its ability to undergo repair is limited [45]. The architectural intricacies highlight the significance of considering the hierarchical structure of articular cartilage in the context of osteochondral research.

### Structure of subchondral bone

The subchondral bone contributes to joint stability and provides structural, mechanical, and nutritional support [46]. It is anatomically divided into subchondral cortical plate and subchondral trabecular (cancellous) bone [47]. Subchondral cortical bone is a thin layer lying immediately underneath the calcified cartilage and is responsible for mechanical support (Fig. 3) [48]. Beneath that, subchondral cancellous bone is metabolically active and has porosity features that adjust to local mechanical influences [49]. Generally, subchondral bone provides structural support to the joint and contributes to its overall stability preventing bone deformation, collectively creating a robust and flexible system [50]. Any abnormalities or changes in the subchondral bone can significantly affect joint health and function [51]. For instance, in OA, subchondral bone goes through remodeling, and bone spurs (osteophyte) grow on it [52]. Together, the articular cartilage and subchondral bone work in harmony to ensure proper joint function.

## Designing of mini-joint (osteochondral) organoids

Advances in three-dimensional (3D) cell culture methods represent a powerful tool that offers many advantages to 2D systems [53]. Moreover, individual monocultures are not capable of modeling the crosstalk between different tissues that are essential for joint homeostasis [54]. By combining different tissue organoids, such as bone or cartilage organoids in biological models, we can reduce the gap between individual cells and a whole organ, which is important for improving research outcomes [55]. Although there is myriad research about bone and cartilage organoids [56–59], we have limited sources for OC organoids. These systems require a more complex environment to grow two tissues with different compositions and structures. Generally, three different methods are used to engineer these organoids, which involve initiating the culture with either osteogenic medium, chondrogenic medium, or utilizing two different plates simultaneously (Table 1). To develop an effective 3D joint model, it is crucial to choose the right cell source, biomaterial, and other essential factors based on the specific goals of the application. Figure 2 illustrates the cell source and biomaterials for OC organoids.

**Table 1:** Developed organoids

References	Cell Courses	Scaffold	Novelty	Methods	Potential Application	Advantage	Disadvantage
Muraglio et al. [64],	Human BM-MSCs	Scaffold-free	Dual chondro-osteogenic differentiation in the same micromass culture	Dependent, initiated with chondrogenic medium	Molecular determinants of endochondral bone	Spatial and temporal pattern of chondro/osteogenesis	The cartilaginous region is surrounded by a calcified shell,
Limraksasin et al. [87],	Murine iPSCs	Scaffold-free	The Effect of Mechanical Stimuli, Self-Organization of Osteochondral tissues directly from iPSC embryonic bodies	Dependent, initiated with osteogenic medium	Osteochondral development	Easier manipulation of bone/cartilage ratio, bony core and cartilaginous shell	Lack of in-vitro experiments
Hall et al. [61],	human PDCs for the bony part and human iPSCs for the cartilaginous part	PDMS	Organoid self-assembly into zoned implant	Two independent separate medium	In vivo functionality, and Endochondral ossification	Using pre-programmed living building blocks, Zone-Specific Functionality, In vivo implantation,	Predifferentiation of the different cell sources, unstable dual maturation
O’Connor et al. [88],	Murine iPSCs	Scaffold-free	The time-dependent study, encapsulation within a 3D matrix prevents the reinduction of pluripotency in differentiated iPSCs.	Dependent, initiated with chondrogenic medium	Endochondral ossification, joint disease, and drug screening	in vitro modeling of specific genetic variants as risk factors for joint-related diseases	The cartilaginous region is surrounded by calcified shell
Abraham et al. [93],	Human pediatric donor Joint tissue	Matrigel	Self-assembling skeletal organoid system derived from human tissue	Two independent separate medium	Tissue development, joint disease, and drug screening	Accessible from small amounts of human tissue, Long-term expansion period, Generation of mini-joint cultures	Lack of endochondral, and OA-derived cell source
Li et al. [80],	Patient-derived iPSCs	Bioceramic	Mimicking intramembranous ossification and articular tissues	Dependent, initiated with osteogenic medium	Developmental mechanism, joint disease and drug screening	Bony core and cartilaginous shell, personalized organoid,	Limited nutrient diffusion to the bottom of the organoids, Lack of in-vitro experiments
Van Hoolwerf et al. [153],	Human iPSC-derived MSCs	Scaffold-free	Evaluate a particular mutation of human Subchondral bone turnover & cartilage mineralization	Dependent, initiated with chondrogenic medium	Genetic mutation underlying subchondral bone turnover and cartilage mineralization in chondrocalcinosis patients	Modeling of specific mutation as risk factors for a joint-related disease	Lack of direct interaction between chondrocytes, osteoblast, and osteoclasts
Yang et al. [66],	Human umbilical cord MSCs	Gelatin Microcryogels	In vitro self-assembly into canine osteochondral defect	Two independent separate medium	In vivo regenerative functionality	Cytocompatibility, cell growth potential, proper interactions with biomolecules and drugs, and simultaneous regeneration of cartilage and subchondral bone in hierarchical layers	The small sample size for in-vitro implantation, insufficient Time to demonstrate full defect repair in canine model, pathological differences between the canine and human

BM: bone marrow, MSCs: mesenchymal stem cells, iPSCs: induced pluripotent stem cells, PDCs: periosteum-derived cells, PDMS: Polydimethylsiloxane

### Cells for osteochondral organoids

The phenotype of the cell source involved in the formation of both articular cartilage and the subchondral bone is intricately linked to the osteochondral organoid development [60, 61]. Pluripotent stem cells (PSCs), such as induced pluripotent stem cells (iPSCs), as well as progenitor cells, and adult stem cells (ASCs), can produce organoids (Table 1) and possess the ability to generate bone, cartilage, or/and osteochondral tissue [62, 63]. All of these cell types have their advantages, and choosing the cells depends on the purpose and application of the organoid.

#### Mesenchymal stem cells

To begin with, several research groups proposed various approaches for mesenchymal stem cells (MSCs) differentiation into bone tissue [64–66]. In native bone microenvironments, MSCs are recruited to form osteoblasts [22] and they can be utilized in vitro studies to give rise to the bony part of organoids. MSCs’ advantageous characteristics such as stemness [67], proliferation [68], and differentiation capacity [69] allow researchers to use them, and their anti-inflammatory [70], and antiapoptotic abilities [71] can make them compatible options for bone organoids. For instance, following the implantation of organoids, the present inflammatory signals polarize MSCs towards an anti-inflammatory and pro-trophic phenotype to aid in tissue recovery [72]. These cells can suppress the expression of genes that promote cell death and contribute to their therapeutic effects [73]. Moreover, they are entirely able to produce and release critical growth factors and cytokines [74]. In addition, the cartilaginous section of OC organoids can be generated from MSCs [75]. They are capable of differentiating into specialized cells developing from mesoderm such as cartilage [76]. These cells are present in multiple tissues, and their chondrogenic potential advances cartilage tissue engineering [77].

The first OC organoid strategy was developed from human bone marrow derived-MSC (BMSCs). They were initially micromass cultured for four weeks. Transforming growth factor-beta (TGFβ1), dexamethasone, and ascorbic acid were used for differentiating MSC. The resulting structures, known as “cartilage beads,” had hyaline cartilage characteristics. Moreover, culturing MSCs in a mineralization inductive medium, successfully resulted in a mineralized bone-like collar around the cartilage. Considerable calcification was a consequence of synthesized collagen type I (COL1), sialoprotein, and osteocalcin (OCN) [64]. Moreover, MSCs-loaded scaffolds demonstrated an osteoconductive environment favorable for bone healing [78]. In particular, loaded umbilical cord MSCs-biomaterial were used in a more recent strategy to form both cartilage and bone in two separate dishes spontaneously. Upregulation of multiple osteogenesis signaling pathways confirmed the commitment of MSCs to osseous lineage and their efficient regulation of mineralized microenvironment [66]. Moreover, MSCs displayed the higher relative gene expression of collagen type II (COL2) and SRY-Box Transcription Factor 9 (SOX9) in 3D cultures [66].

MSCs produced from iPSCs also hold great generative potential for joint-related disorders, showing promise in OC repair [79]. In another research, iPSC-derived MSC was first cultured in an osteogenic environment and afterward, maintained in the cartilaginous medium for 21 days to promote cartilage development on the surface. The chondral outer region of the osteochondral organoid exhibited abundant deposition of COL2, similar to the superficial zone. However, compared to the cartilage organoids, the co-cultured OC organoids demonstrated lower expression of aggrecan (ACAN), and the levels of COL2 and SOX9, were just the same [80].

#### Induced-pluripotent stem cells

There has been a growing focus on iPSCs due to the limitations associated with MSCs in terms of their regenerative capabilities [81]. MSCs have been used in some clinical bone regeneration. However, they have critical shortcomings, such as heterogeneity, differentiation potential, and migratory capacity [82, 83]. An alternative approach for generating in vitro OC models involves using iPSCs [84]. These cells are able to create unlimited cell sources for bone and cartilage regeneration and maintain the genetic background [85]. As mentioned before, in embryonic development, bone and cartilage rise from mesodermal origin. Therefore, in some approaches, recapitulating an intermediate step to generate mesodermal cells is necessary [86].

There are two research that applied mice iPSCs to develop organoids successfully [87, 88]. In the first one, Limraksasin et al. used a stepwise protocol, beginning with the administration of trans-retinoic acid to iPSCs to achieve mesodermal lineage commitment. In the next step, the pre-somatic mesoderm was differentiated into osteoblast via an osteogenic growth medium in a 3D sphere culture. After 10 days, the medium was replaced with a chondrogenic one and maintained for 21 days. The former medium results in the development of some cartilage-like tissue, which stimulates both osteogenesis and chondrogenesis gene expression. Cultivation in the later medium leads to a substantial area of cartilage tissue, with a significant increase in the chondrogenic gene expression. This induction additionally enhanced the commitment of mesodermal lineage, as demonstrated by the sequential expression of mesoderm marker genes. The cartilage-like tissues primarily emerged in the exterior layer, where a cluster of cells with chondrocyte morphology were found in lacunae. Manipulation of the induction protocol can alter the bone-to-cartilage ratio in model [87]. This indicates that iPSC-derived cells were not restricted to bony fate and maintained their potential to transdifferentiate in the cartilage pathway, which was confirmed in another study [80]. In contrast, the O’Connor research group initiated their strategy with chondrogenic induction of murine iPSCs by differentiating them in micromass culture and subsequently cultured in chondrogenic media for 45 days. These cells produced a cartilaginous matrix with s-GAGs and Col 2 and 6 that remained in the center of the organoids. They also demonstrated chondrogenic gene expression including Acan, Col 2, proteoglycan 4 (Prg4), and Sox9. Subsequently, the cell pellet was cultured in osteogenic media for 28 days [88]. Through this method, mature chondrocytes were triggered to differentiate into osteoblasts which consistently indicated the long-term potential of their iPSC source [89].

Moreover, human iPSC-derived chondrocytes could shape cartilage microtissues and form zonal structures [61]. Some articular cartilage-associated mRNA expression levels were significantly higher than the bony part of the organoid. However, *SOX9, COL2*, and *COL1* were no different between these two parts [61]. Regardless of the vast opportunities iPSCs offer for cartilage regeneration, their application is limited regarding their expenses and recapitulating vivo functionality [90]. The iPSC-derived organoids are incapable of demonstrating the natural environments and have limitations in self-organizing with their lack of scalability [91, 92]. It is essential for researchers to carefully weigh the benefits and shortcomings of MSCs and iPSCs to reach their goals and obtain superior results.

#### Tissue resident cells

Another potential cell source for generating OC organoid is from joint-resident cells [93]. For instance, Periosteum-derived cells (PDC) can be the origin of osseous sections of organoids and hold noticeable promise for advancing regenerative medicine and tissue engineering applications in the field of orthopedics [61]. Periosteum has a connective bilayer texture that covers the bone surface contains osteoprogenitor cells and is responsible for providing nutrients, osteogenesis, and bone repair [94]. PDCs are involved in osteogenic development, homeostasis, and repair and exhibit strong potential for bone tissue regeneration due to their proliferative and osteogenic differentiation capabilities [95]. The periosteum, located within a mechanically dynamic environment, serves as a specialized microenvironment conducive to the maintenance and proliferation of pluripotent stem cells [96]. In comparison with BMSCs, the periosteum resident stem cells have a larger capacity to repair bone tissue [58]. PDCs-derived organoids have been successfully developed and demonstrated the mineralized part of the osteochondral-like tissue. After 21 days of culturing in a chondrogenic medium, they showed hypertrophic gene markers and formed a microtissue that got implanted and shaped the bony section of the organoid [61]. All in all, these organoids, irrespective of their origin, offer unprecedented means to study osteochondral tissue in vitro.

#### Cell-free osteochondral constructs

Cell-free osteochondral strategies do not contain living cells and are composed of biomaterial scaffolds that are designed to replicate the native extracellular matrix of OC tissue [97]. Due to the challenges of creating artificial biomaterials that reflect the chemical and topographical features of cellular environments [98], there is growing interest in using naturally derived ECM as a biological scaffold. This ECM scaffold is obtained through decellularization, which aims to eliminate native cells and genetic components like DNA and RNA while preserving its biochemical and biomechanical properties [99]. Recellularizing the decellularized ECM with patient cells, makes it possible to generate effective personalized tissues [100]. These scaffolds have been used clinically in various organs and successfully promoted tissue regeneration [101]. Acellular osteochondral ECM should preserve the connection of the bone-to-cartilage border and be affordable and biodegradable to restore OA and other defects [102, 103]. Rowland et al. applied a decellularized scaffold to develop joint organoids in a spatiotemporal controlled condition via site-specific, tunable, and inducible protein delivery systems. This construct serves as a valuable tool platform to monitor inflammatory signaling in osteochondral repair [104]. In addition, in a recent study, an efficient decellularized OC sheet was repopulated by BM-MSCs and demonstrated largely preserved interface integrity between cartilage and bone in the joint structure. Following the implantation of this scaffold, effective cell penetration, proliferation, and differentiation into osteoblasts and chondrocytes occurred, as well as ECM secretion were observed [105]. To achieve optimal results with decellularized tissues, it is essential to carefully control scaffold degradation properties and the simultaneous formation of cartilage and bone. Time plays a crucial role in this process, as the unpredictable degradation of decellularized scaffolds may not provide sufficient time for the development of mechanically competent tissue, especially within the challenging conditions of joint pathology [106].

### Osteochondral targeted biomaterials, biomolecules, and physical factors

In addition to selecting appropriate cells, scaffolds and signaling factors (biochemical, chemo-physical, and physical signals) are crucial in tissue engineering. The generated organoid is expected to have a high resemblance to the natural tissue in chemical, physical, and functional aspects to succeed in research studies [107]. Biomaterials with/without growth factors present promising platforms to get the most out of cells’ capacities by obtaining microenvironments to achieve spatial complexity [108]. The complex hierarchical OC unit needs the application of both bone and cartilage-associated biomaterials for the repair and regeneration of defects.

Although some experiments have been developed in the scaffold-free environment [64, 88], others utilized polymers, bioceramics, and extracellular matrix (ECM)-derived materials (Table 1). Generally, a suitable natural or synthetic scaffold should fulfill these requirements: biocompatibility, bioactivity, protective mechanical strength, the capacity of adherence morphology, proliferation and/or differentiation of the embedded cells, ability to imitate the native ECM, bio-integration, and biodegradability [44, 109]. In addition, designing a scaffold for osteochondral engineering requires osteo-inductivity, osteo-conductivity, and mechanical properties such as appropriate pore size and surface roughness [110, 111]. In the following paragraphs, we will mainly summarize the suitable microenvironment for OC organoid engineering.

#### Bone-associated biomaterials, biomolecules, and physical factors

There are various growth factors associated with bone differentiation and regeneration, including parathyroid hormone-(PTH), insulin-like growth factor (IGFs), platelet-derived growth factor (PDGF), and bone morphogenetic proteins (BMP) [112]. These factors modulate cell migration, adhesion, proliferation, differentiation, and survival [113]. A proper scaffold can facilitate growth factor secretion that triggers the osteogenesis pathway [114]. In a time-dependent strategy, the O’Connor group applied BMP-2, a particular growth factor in the osteogenesis medium [88]. BMP-2 has a regenerative effect on bone defects and is capable of increasing the expression of alkaline phosphatase (ALP), and Runt-related transcription factor 2 (RUNX2). Therefore, it has been used for differentiating stem cells toward osteogenic lineage [115]. BMP2-included scaffolds can be applied to generate in vivo bone-related organoids for destructed bone tissue [116]. However, O’Connor et al. induced an osteochondral organoid in a scaffold and bioreactor-free system. In the final 28 days of the 3D culture, they induced osteogenic media, and their model appeared to have a dense mineralized outer layer rich in COL6. Moreover, the higher expression of OCN, ALP, RUNX2, bone sialoprotein (BSP), and COL1 genes confirmed the existence of the osseous outer [88]. The extracellular matrix is responsible for arranging the local distribution of growth factors by regulating their concentration and duration. Hence, producing a suitable matrix is vitally necessary for bone tissue engineering [117].

Bioceramics are one of the most practicable materials in bioengineering methods. They are well-known for their oxidation resistance, high mechanical strength, and biocompatibility [118]. Due to their porous structure, they can easily integrate with bone and due to their osteo-inductive properties can be utilized to recover osteochondral defects optimistically [119]. For instance, Li et al. used hydroxyapatite nanorod (HANR) which is an osteo-inductive bioceramic nanoparticle to produce OC organoid model [80]. This particular nanomaterial is synthesized from calcium hydroxide and ortho-phosphoric acid. It bears a resemblance to the mineral parts of bone tissue and is an effective substance in bone tissue engineering [120]. After treating cells and their produced ECM with Ascorbic acid, HANRs were added to induce osteogenesis for 21 days in a vitamin D3-contained medium. This HANR-included ECM helped them to generate a highly mineralized bony core and cartilage shell that showed higher level expression of bone-associated proteins including ALP, OCN, and RUNX2 which confirmed the increased osteogenesis efficiency of HANR-containing matrix [80].

Gelatin-based microcrystal is another biomaterial used in generating osteochondral organoid strategies. Gelatin which is a natural collagen-derived biopolymer, is widely used in tissue engineering, through diverse strategies, and supports cell growth with its biodegradability and biocompatibility [121]. Microcryogel is a small-scale scaffold that benefits organoid engineering by providing 3D microniche to load cell and growth factors and is utterly practicable in cell therapy due to its capability to get injected [122]. Various cells can get loaded on the microcryogel to generate a cell-laden construct with an oriented differentiation pathway [123]. Their structure enhances self-assembly toward a prearranged shape in 3D culture. This porous material appeared to support the stemness of MSCs, improve their secretion, reduce their senescence, and enhance cell-ECM interaction [124]. Yang et al., predifferentiated the microcryogel via hydroxyapatite (HYP) to develop osteogenic (OS) microcryogel. Their model demonstrated sufficient cell proliferation, interaction surface, and cytocompatibility. First, they seeded the cells on the microcryogel and induced differentiation via the osteogenic medium. Then, customized a meshed frame with defined space and loaded the OS-microcryogel at the bottom layer. Increased ALP, RUNX2, and calcium deposition affirmed the potential of OS-microcryogel in improving MSC differentiation. It also provides an environment for several blood vessels to grow, unlike chondrogenic-microcryogel. The organoid demonstrated correct interactions and cytokine secretion in vivo. This scaffold was superior to growth-factor-based methods in some aspects: the porous composition, cell viability, function protection, and fitting the defect size due to its small size [66].

Physical force is another compelling element in the OC organoids generation process. The osteochondral unit is subject to mechanical pressure [125]. Particularly, cartilage is frequently exposed to various mechanical forces, such as tension and shear stress [126]. Limraksasin et al. used an ultra-low attachment micro space plate to induce osteogenesis by subjecting it to shaking force. Physical force positively impacted osteogenesis and cell condensation, facilitating the self-organizing process of cells to form the organoid. This structure features a calcified inner region surrounded by a rich osteoblastic layer containing COL I [87].

#### Cartilaginous-associated biomaterials, biomolecules, and physical factors

In harmony with bone differentiation, the natural cartilaginous microenvironment requires various growth factors. These biomolecules orchestrate the pivotal pathways responsible for chondrogenic proliferation, differentiation, and apoptosis [127]. TGFs-β family is one of the effective growth factors in chondrogenic development. A combination of this growth factor with micromass, a 3D culture that provides a chondrogenic environment similar to embryonic development, has been suggested for studying chondrogenesis [128]. Accordingly, in a study by O’Connor, a scaffold-free micromass environment was utilized to form iPSC cells pellet and after digestion, the chondrogenic medium was added that contained TGF-β3 ^88^. The chondrogenic center of the organoid was rich in sulfated glycosaminoglycans (s-GAGs) and COL2 and had activated pathways of ACAN; a resistant factor to compressive loads [129], proteoglycan 4 (Prg4); a joint/boundary lubricant [130], and Sox9; a major chondrocyte transcription factor [131]. In addition, their chondrogenic matrix was resistant to the pluripotent state and remarkably prevented cell reprogramming pathway [88]. Notably, the 3D chondrogenic culture environment demonstrated a lower capacity for undergoing osteogenic differentiation [66] highlighting the prominent role of matrix in preventing the reinduction of differentiated iPSCs. However, unlike the native structure, the shelly region in this study was observed in the center of the construct.

Alternatively, several research groups utilized scaffold materials that facilitate the regenerative capacity of cartilage. A suitable scaffold for cartilage cultivation should possess proper physical properties; stiffness, bio integration, flexibility, structural features; porosity, permeability, and functional traits; adhesion, proliferation, and differentiation capability [23, 55]. Furthermore, a 3D scaffold provides an environment for cartilage to produce and secrete the necessary cytokine and other proteins, and it also prevents dedifferentiation to fibroblast-like cells [132]. These biomaterials can be both natural and artificial, and each of them has its advantages and disadvantages. Natural ones such as hyaluronic acid (HA) have similarities to native tissue environments [133]. However, they have some limitations, including inflexibility, time-limited functionality, and low stability [134]. Therefore, synthetic scaffolds showed conspicuous efficiency. As mentioned earlier, HANR-included culture was used in a study to generate OC organoids. In combination with a cartilage-mediated medium that contained BMP6 and TGF- β3, subsequently, they led to a chondral shell in the last 21 days of cultivation. The exterior region appeared to have a high level of COL2 and GAG [80]. However, fabricated scaffolds may have insufficient biological properties and unexpected breakdowns. Consequently, this highlighted the superiority of combined natural and synthetic biomaterials [134].

To use both of these scaffolds, loaded umbilical cord MSCs-biomaterial were used in a more recent strategy to spontaneously form both cartilage and bone in two separate dishes. Yang et al. designed their experiment by mixing gelatin and 6% hydroxyapatite (HA) to fabricate a microcryogel suitable for chondrogenesis, that appeared to be effective in cell adherence, differentiation, and survival. After seeding the cells and embedding them on a poly (lactic-co-glycolic acid)/gelatin scaffold, the chondrogenic differentiation process began by using a TGF-β-included medium. This scaffold specifically provided a chondrogenic-specific environment that allowed cells to secrete GAG and express a sufficient level of COL2 and SOX9 and averted the osteogenic markers expression [66]. Nevertheless, the biophysical and biochemical aspects of organoid models, affect their functional effectiveness and their resemblance to native tissue.

## Application of osteochondral organoids

**Fig. 4:**
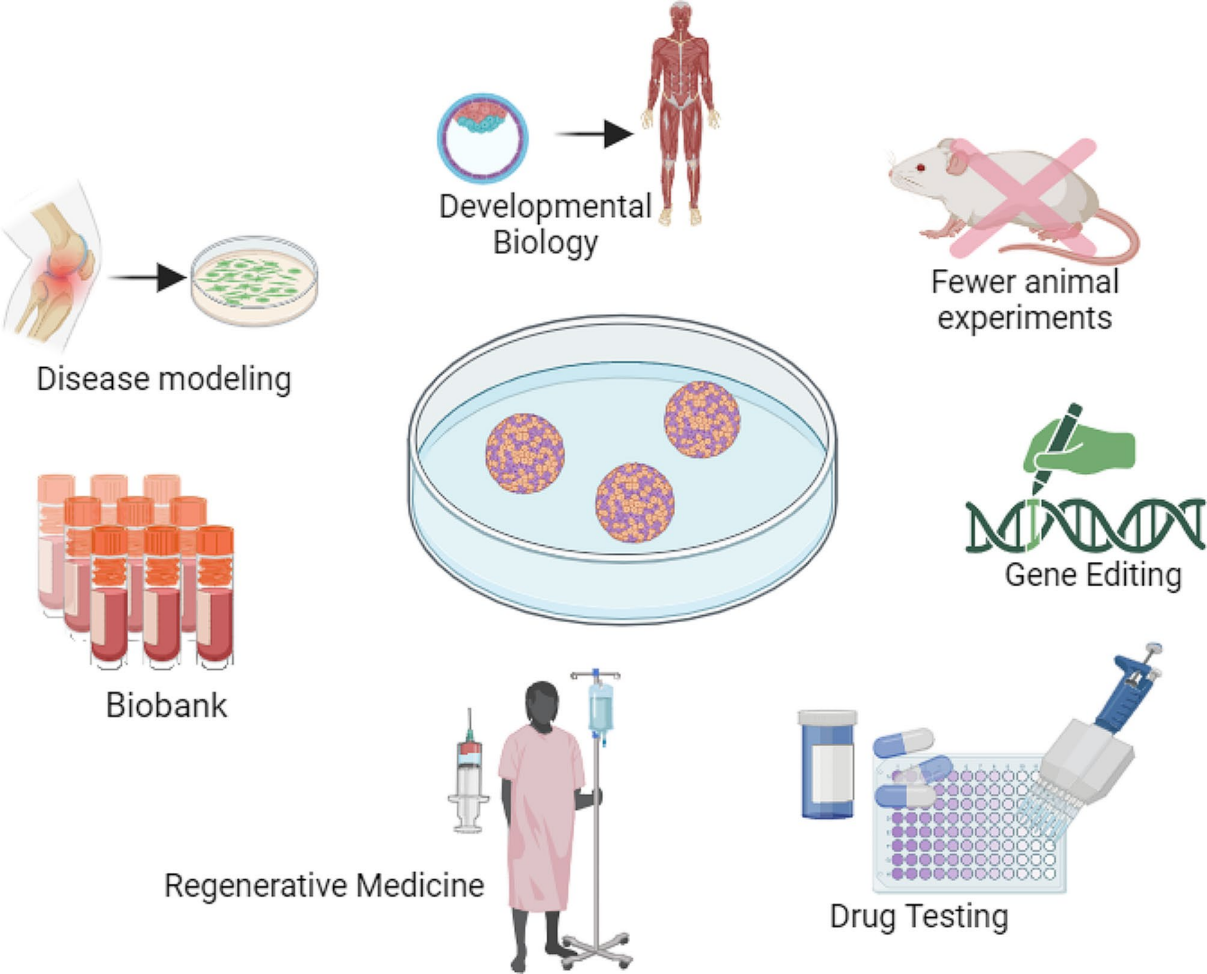
The schematic figure shows the osteochondral organoids advantages

Organoid cultures provide tremendous advantages, including the ability to generate from both healthy and diseased cell sources [135]. They can be expanded over extended periods, ensuring to preservation of their genetic stability [136, 137]. Furthermore, these cultures can be cryopreserved to generate biobanks for future research [138]. Compared with 2D culture, 3D organoids have more resemblance to physiological conditions and provide a platform to manipulate signaling pathways and perform genome editing [139]. As such, these cultures have been used for various applications including drug discovery, developmental biology, personalized diagnostics, and cell therapy (Fig. 4).

### Study of bone and cartilage development and bone–cartilage crosstalk

3D cell-cultured methods are superior to animal and 2D models in various aspects. They can shape diverse cell types in a complex microstructure, allowing for demonstration of the cell-cell and cell-microenvironment interaction in all three dimensions [140]. In addition, manipulating the defined gradient concentration of growth factors, cytokines, essential nutrients, and waste products is more pragmatic in 3D cultures, compared to 2D monolayer [141]. Osteochondral organoids elucidated the molecular biology involved in the development, thus offering a comprehensive framework for studying the underlying mechanisms of articular cartilage and OC joint.

Several research investigated the crosstalk between bone and cartilage and their development through tissue-engineered approaches [142–144]. As we mentioned before, the cells and their produced messenger biomolecules such as growth factors affect other cells and their microenvironment in the complex joint structure. Therefore, almost all studies could directly/indirectly demonstrate the interaction between bone and cartilage. For instance, in Limraksasin’s study, not only the initiation osteogenic medium induced the osteogenic part, but also chondrogenic induction of iPSC enhanced the osteogenic markers such as Col1 and Osterix (Osx). Moreover, they demonstrated that endochondral ossification is regulated by some critical transcription factors, including both Sox9 and Osx [87]. Endochondral ossification is one of the most studied processes describing bone formation [58]. O’Connor et al. demonstrated the natural progression of the cartilage-to-bone interface during development in iPSC-derived organoids successfully. They illustrated that mature chondrocyte cells directly differentiate into osteocytes and osteoblasts to create bone tissue [88]. However, the complex molecular and cellular mechanisms may be further inquired to clarify endochondral ossification and other interactions.

### Study of diseases models

The use of disease-specific organoids will facilitate the analysis of the cascade of molecular, cellular, and biomechanical signals and seek new treatments for degenerative joint diseases to improve patient care and outcomes [145]. Cartilage degradation, inflammation, and joint stiffness can be studied in 3D cultures [146]. 3D models also have the potential for exploring patient-specific genetic risk factors [88]. Disease-specific organoids can help to identify promising novel therapies and provide patient-derived platforms for drug screening that shed light on personalized medicine [147]. As mentioned earlier, OA is one of the most studied joint-related diseases, thus, generating OA organoids can be beneficial in the orthopedic field. For instance, Abraham et al. harvested diseased cells to study OA pathobiology and evaluate its potential treatments [93]. It is noteworthy that Interleukin-1β (IL-1β) is widely used as a pro-inflammatory cytokine to induce most joint diseases [148]. It is involved in cartilage destruction and inhibition of chondrogenic ECM formation in OA [149].

These models facilitate investigating diseases in numerous aspects. To illustrate, microRNA signaling is one of the alterations associated with OA progression [150] and their dysregulation can be studied in OA organoid models [151]. The advances in genetic engineering enable understanding of multiple biological phenotypes through 3D models [152]. Van Hoolwerff et al. studied mutation of genes encoding osteoprotegerin which is a critical protein in OA and their potential as hallmarks of this disease. They utilized organoid models to show that the mutations can directly affect chondrocytes and osteoblasts [153].

### Drug testing programs

Animal models and 2D cell cultures have been used to deepen our knowledge of joint-related disorders, disease-modifying OA drugs (DMOADs) discovery, and to assure safety before clinical trials with human subjects [154]. However, due to the intrinsic species differences between human and animal models and ethical concerns, several obstacles appeared in testing novel drugs, investigating the metabolism pathway, and examining side effects [155]. 2D cell cultures are unable to recapitulate the heterogeneity of in vivo disease and unable to represent the in vivo physiological condition [156]. Therefore, organoid technology evolved as a potential approach to facilitate drug testing process [157]. Pharmaceutical companies can utilize 3D organoids for drug screening, as well as for evaluating drug metabolism, toxicity, and side effects [158]. This approach enables the delivery of precise data and facilitates the adaptation of studies for high-throughput performance [159]. Moreover, patient-derived organoids, with their maintained genetic heterogeneity, are superior platforms for personalized evaluation [160]. A shorter detection cycle, lack of organ toxicity, and cost-efficiency are some advantages of screening drugs on these cell cultures [161].

Tissue-engineered models broaden new opportunities for customized drug validation of genetic disorders [162], inflammatory diseases [93], and cancer [163]. Abraham et al. developed an organoid for testing an anti-inflammatory agent as a regenerative therapy for OA. The effects of Adenosine A2A receptor (A2AR) agonist were evaluated in an OA organoid. Although it successfully upregulates two transcription factors that reduce inflammation, it could not enhance differentiation and regeneration [93]. The recent development of DNA nanostructures can progress novel drug design and delivery systems with remarkable editability and biocompatibility features and may improve OC organoids [66]. There is a greater emphasis on utilizing bone or cartilage organoids for drug screening [164, 165]; however, the number of studies exploring osteochondral organoids as platforms for drug testing remains limited in the scientific literature. The various techniques used for organoid production have developed in very recent years, and further improvements are required to advance the accuracy, precision, and efficiency of drug monitoring of osteochondral-related diseases.

## Osteochondral chip models

Organoid-on-a-chips are miniature systems that mimic the physiological and functional aspects of a particular organ/tissue by controlling tissue-specific microenvironments such as fluid flow, the culture condition, and interactions [163, 166, 167]. With the mechanical stimuli and bioactive cues, these models can be superior to organoids due to their high controllability. Biosensors, fabrication material, proper scaffolds, and cell sources assisted researchers in generating organoid-on-chips [168]. Chips-based joint models can be applied to advance our knowledge of joint pathology and the progress of promising novel treatments [169, 170]. Therefore, demonstrating OA phenotype and evaluating DMOAD in these systems is expected as ordinary (Table 2). In one study, iPSCs surrounded with gelatin scaffolds in a dual-flow bioreactor, and consequently, the generated OC chips faced IL-1 𝛽 treatment to show OA condition. This provided a real circumstance to learn the crosstalk between bone and cartilage along with screening Celecoxib, a commonly prescribed drug [171]. Additionally, MSCs have been used to engineer a more complex construct: osteochondral among other tissues including, adipose and synovial-like fibrous. A methacrylate gelatin hydrogel scaffold was applied to create a 3D environment and the efficiency of Naproxen and four underdeveloped drugs, including fibroblast growth factor 18, IL-1RA, sclerostin, and SM04690 were tested for OA treatments [170]. Although various chip models appeared as potential drug screening applications, they are still incapable of recapitulating the exact physiology of the natural tissues in mechanical studies [1].

**Table 2:** Joint related Chip Models

References	Cell Sources	Scaffold	Generated Tissue	Potential and Advantage
Shi et al. [201],	hAMSCs	Hydrogel	Osteoblasts, Chondrocytes,	Biomimetic Bone-to-Cartilage Interface
Lin et al. [202],	hBMSCs	Methacrylate Gelatin scaffold	Osteochondral Tissue	Joint Physiology, OA Pathology, DMOAD Screening
Lin et al. [171],	human iPSCs	gelMA Scaffolds	Osteochondral Tissue	Joint Physiology, OA Pathology, DMOAD Screening
Rothbauer et al. [169],	FLS and Human Primary Chondrocytes	Hydrogel	Synovium and cartilage	Tissue-level Cross talk, Patient-Derived RA Model
Mondadori et al. [203],	Synovium, Articular Cartilage, HUVEC, OA-derived Human Primary Monocytes	Hydrogel	Synovium, Cartilage, Endothelial Monolayer, Monocytes	Tissue-level Cross talk, Patient-Derived OA Model, Vascularized Tissues, OA and RA Pathology,
Tuerlings et al. [204],	Primary Chondrocytes and Osteogenic Cells	PCL-based scaffolds	Osteochondral Tissue	OA Pathobiology and Drug Screening
Pirosa et al. [205],	hBMSC and HUVECs	PCL/hydroxyapatite (HA) scaffolds combined with gelMA hydrogel	Osteochondral Tissue	Triphasic Vascularized Osteochondral Tissue Interface
Li et al. [170],	hBMSC	Hydrogel	Osteochondral, Synovial-like fibrous, and Adipose tissue	OA Pathology, and Drug Screening

PMMA, polymethyl methacrylate; hAMSCs, human adipose-derived mesenchymal stem cells; hBMSCs, human bone marrow stem cells; iPSCs, induced pluripotent stem cells; OA, osteoarthritis; DMOADs, disease-modifying OA drugs; FLS, Fibroblast-like synoviocytes; gelMA, methacrylate gelatin; RA, rheumatoid arthritis; HUVEC, Human Umbilical Vein Endothelial Cells; PCL, polycaprolactone HA, hydroxyapatite

## Challenges of osteochondral organoids

While OC organoids simulate some of the critical aspects of the joint, their use in biomedical applications on a large scale is still limited by our current inability to fabricate a functional and structural unit of OC, maintain scalability, and cost-effectiveness as much as their safety [172–174]. Their application in biomedical treatments depends on organoid size, shape, cell composition, and survival. The generation of an osteochondral unit with the seamless gradient of the bone part containing nerve, blood vessel, and mineralized ECM, and a cartilaginous part as aneural, avascular, and non-mineralized, is not controllable. Managing these aspects to reach the optimal condition may be challenging, considering the time element and the value of long-term preservation [175].

Mimicking the whole osteochondral unit with its diverse cell sources such as osteoblast, osteocyte, chondrocyte, synoviocytes, and the microenvironment is complicated [176]. On other words, the simultaneous differentiation of cells in bony and cartilaginous parts of organoids using a set of different cells, biomaterials, and bioactive factors is noncontrollable. Furthermore, three-dimensional organoids often lack essential organ-specific cells, such as tissue-resident macrophages that play crucial roles in the immune responses against infections and diseases [177, 178]. Enhanced homeostatic mechanisms of macrophages can be used as a long-lasting treatment for OA [179]. Therefore, their presence in organoid models can be beneficial. However, current methods lack communication between the immune and musculoskeletal systems which is crucial for regulating tissue regeneration [180].

In vivo tissue-engineered grafts show limited capacity to regenerate the damaged tissue due to poor integration with host cartilage and the failure to retain structural integrity after insertion, resulting in reduced mechanical function [181, 182]. Moreover, they are not capable of achieving the same complexity of the interfacial tissue size and gradient structure as native organs and lack the crucial directional cues and physical, structural, and mechanical properties [183]. The mechanical properties of articular cartilage are highly divergent in different layers, and recapitulating this complexity is effortful [184].

The limited self-repairing capacity of cartilage makes restoration of its mechanical properties challenging [185]. Lack of certain zones impairs load-bearing capacity, affects biomechanical properties, and impedes joint health [186].

As previously indicated, specific existing techniques for organoid generation rely on costly growth factors, making large-scale production prohibitively [187]. Their short half-life, expensive costs, and weak portability limit growth factors’ practicality in organoid development [188]. Additionally, some methods necessitate the incorporation of engineered biomaterials to establish controllable conditions. These strategies require scaffolds that mimic the native architecture and function precisely [189]. In other words, the main challenge is to determine the combination of different scaffolds, cells, and biomaterials that perfectly create an OC microenvironment that enhances tissue growth and closely mimics the native tissue environment. However, researchers face challenges in monitoring and controlling every aspect of the development or/and implantation processes to develop similar organoids to natural tissues [190]. It is noteworthy that future ethical research is required to study organoid implantation in humans [191]. Furthermore, generating vascular networks within osteochondral organoids to support nutrient and oxygen diffusion throughout the structure is a critical challenge that needs to be addressed for long-term viability [192]. Overcoming these technical limitations can make organoid technology a remarkably effective biomedical clinical tool.

## Conclusion and perspective

The inaccessibility of in vivo human samples and differences between animal models and human biology are the noticeable obstacles in studying joint development and diseased states [193]. The development of 3D organoids requires suitable cell origin, effective biomaterial, and controlled conditions. The specific type of model created may vary depending on the desired application and the researchers’ goals. Although these models offer several advantages, some drawbacks need to be addressed.

By subjecting osteochondral organoids to controlled mechanical stimulation, tissue maturation can be improved, and the development of physiologically relevant mechanical properties can be promoted [194]. Improvement of nutrient and waste exchange within osteochondral organoids directly affects their survival [195]. Therefore, establishing vascularization strategies, such as incorporating endothelial cells or bioactive factors, can improve the functionality and viability of organoid models [196].

Creating a multi-organoid platform that offers high physiological and clinical relevance for comprehensive mechanistic studies and preclinical assessment of potential DMOADs and disease-modifying antirheumatic drugs (DMARDs) can be a promising approach for the most common joint-related disease, OA and RA. A practical method to link these organoids and facilitate their mutual communication is through their integration into an organoid-on-a-chip system or co-culturing [170]. This can enhance the mimicry of native osteochondral tissue and promote cross-talk between different cell populations [197]. Moreover, using advanced biomaterials as well as technologies can provide a conducive microenvironment for osteochondral organoid development and maturation [198]. For instance, leveraging bioprinting technologies can precisely pattern multiple cell types and extracellular matrix components with tunable properties that create biomimetic osteochondral organoids [199, 200].

In conclusion, osteochondral organoids offer enormous promise in advancing our understanding of OC tissue development, disease mechanisms, and therapeutic application. They have the potential to revolutionize the field of musculoskeletal research and contribute to improved treatments for joint-related disorders such as osteoarthritis and cartilage injuries. Further research is required to generate physiologically relevant osteochondral organoids that are operational in regenerative medicine.

## Data Availability

Not applicable.
